# Voltage-Activated Calcium Channels as Functional Markers of Mature Neurons in Human Olfactory Neuroepithelial Cells: Implications for the Study of Neurodevelopment in Neuropsychiatric Disorders

**DOI:** 10.3390/ijms17060941

**Published:** 2016-06-14

**Authors:** Héctor Solís-Chagoyán, Edgar Flores-Soto, Jorge Reyes-García, Marcela Valdés-Tovar, Eduardo Calixto, Luis M. Montaño, Gloria Benítez-King

**Affiliations:** 1 Laboratorio de Neurofarmacología, Instituto Nacional de Psiquiatría Ramón de la Fuente Muñiz, Calzada México-Xochimilco No. 101, Col. San Lorenzo-Huipulco, Mexico City 14370, Mexico; hecsolch@imp.edu.mx (H.S.-C.); mvaldes@imp.edu.mx (M.V.-T.); 2 Departamento de Farmacología, Facultad de Medicina, Universidad Nacional Autónoma de México, Mexico City 04510, Mexico; edgarfloressoto@yahoo.com.mx (E.F.-S.); reyes.garcia.jorge@gmail.com (J.R.-G.); lmmr@unam.mx (L.M.-M.); 3 Departamento de Neurobiología, Instituto Nacional de Psiquiatría Ramón de la Fuente Muñiz, Calzada México-Xochimilco No. 101, Col. San Lorenzo-Huipulco, Mexico City 14370, Mexico; ecalixto@imp.edu.mx

**Keywords:** calcium flux, differentiation process, human precursors, neuronal excitability, voltage-gated Ca^2+^ channels, neuropsychiatric disorders

## Abstract

In adulthood, differentiation of precursor cells into neurons continues in several brain structures as well as in the olfactory neuroepithelium. Isolated precursors allow the study of the neurodevelopmental process *in vitro*. The aim of this work was to determine whether the expression of functional Voltage-Activated Ca^2+^ Channels (VACC) is dependent on the neurodevelopmental stage in neuronal cells obtained from the human olfactory epithelium of a single healthy donor. The presence of channel-forming proteins in Olfactory Sensory Neurons (OSN) was demonstrated by immunofluorescent labeling, and VACC functioning was assessed by microfluorometry and the patch-clamp technique. VACC were immunodetected only in OSN. Mature neurons responded to forskolin with a five-fold increase in Ca^2+^. By contrast, in precursor cells, a subtle response was observed. The involvement of VACC in the precursors’ response was discarded for the absence of transmembrane inward Ca^2+^ movement evoked by step depolarizations. Data suggest differential expression of VACC in neuronal cells depending on their developmental stage and also that the expression of these channels is acquired by OSN during maturation, to enable specialized functions such as ion movement triggered by membrane depolarization. The results support that VACC in OSN could be considered as a functional marker to study neurodevelopment.

## 1. Introduction

The physiopathology of schizophrenia (SZ) has been associated with alterations in the fetal brain development in the second trimester of pregnancy. In particular, abnormal neurogenesis, neuronal migration and differentiation take place causing structural brain alterations and diminished synaptic contacts. Evidence supporting the neurodevelopmental hypothesis in the etiology of various neuropsychiatric disorders has been reported. In patients with SZ, histopathological analysis of post-mortem brain samples has revealed alterations in the proliferation of hippocampal neuronal precursors [1,2]. In addition, the expression of immature markers predominates in this brain structure in SZ [3,4], and the ratio of mature/immature olfactory sensory neurons is reduced [5], suggesting an impairment in neuronal maturation. Furthermore, a diminished odor perception and a decreased volume of the olfactory bulb have been described in neuropsychiatric patients [6]. One important feature is that odors are sensed at the olfactory neuroepithelium, through specialized receptors coupled to adenylate cyclase III and calcium channels [7]. This neuroepithelium, located at the nasal cavity, is also rich in neuronal precursors that can be collected by non-invasive procedures such as exfoliation [8], and offers a reliable model to characterize and study the neurodevelopment process *in vitro*, in samples obtained from healthy subjects or from patients with neuropsychiatric disorders. For in-depth studies with this model, it is necessary to establish the similarities between neurons from the Central Nervous System (CNS) and from the olfactory neuroepithelium at specific neurodevelopmental stages.

In adulthood, the ability to supply newborn neurons continues in several neurogenic structures of the CNS, which enables the replacement of dead neurons [9,10] in order to maintain the integrity of the cerebral architecture and its functioning [11]. The integrated-newborn neurons originate from precursor cells capable of differentiating and specializing into neuronal or glial lineages [12,13]. Precursor cells from several neurogenic zones in rodents have been isolated and maintained under culture conditions [14]. *In vitro*, the mechanisms of excitability to generate electrophysiological signals (including VACC) are only present in cells that express molecular markers that correspond to mature neurons, and these properties are absent in undifferentiated neuronal precursor cells [15,16], suggesting that these mechanisms are acquired along the neuronal differentiation.

Similarly, in the olfactory neuroepithelium, OSN are continuously replaced even in adulthood [17]. In OSN, VACC are involved in the olfactory pathway by which the chemical properties of odorants are transduced into electrophysiological signals to encode them in distinctive patterns of action potentials [7]. In this peripheral neuroepithelium, as well as in neurogenic areas of the CNS, replacement of dead neurons and glial cells depends on the differentiation of mitotically active precursor cells [18]. Isolation of neuronal precursors from the human olfactory epithelium has been performed by biopsy, laser-capture microdissection, or exfoliation [8,19,20,21], and the enrichment of the culture with mitotically-active precursor cells has been performed by the cloning assay which allows for obtaining robust cellular responses to pharmacological and physiological stimuli [22]. Because the culture of human olfactory epithelial precursors maintains a population with an undifferentiated state but also some of the cells might mature spontaneously into neurons, we therefore hypothesized that this culture constitutes an adequate experimental model to determine whether the expression and functioning of VACC differ among neurodevelopmental stages.

Before addressing the study of VACC in cells from neuropsychiatric patients, in this work, we characterized VACC in cloned cells from the olfactory neuroepithelium of a healthy subject. The presence of VACC at specific developmental stages was detected by immunofluorescence staining, and the functioning of VACC was determined by microfluorometry and electrophysiological recording. VACC were co-detected with Olfactory Marker Protein (OMP) in OSN, but not in nestin(+) cells (neuronal precursors). OSN responded to forskolin with a high entry of Ca^2+^, and depolarization steps evoked a specific Ba^2+^ inward current, whereas in precursor cells, the forskolin-induced response was lower and Ba^2+^ currents were absent. These results strongly suggest that expression of functional VACC is a mechanism of excitability acquired by OSN along their differentiation, and therefore could be used as a marker of functional maturity of these neurons.

## 2. Results

### 2.1. Antibody Characterization by Western Blot

**Figure 1:**
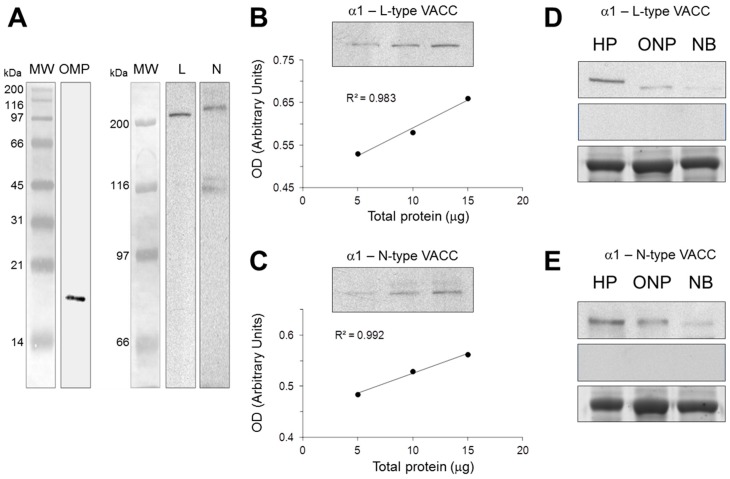
Characterization of OMP and VACC antibodies. Specificity of antibodies was assessed by Western blot, using rat olfactory epithelium extracts for OMP detection, as well as mouse hippocampus (HP), cultured human olfactory neuronal precursors (ONP) or neuroblastoma N1E-115 cell (NB) extracts for VACC detection. (**A**) Antibodies against OMP, and *L*- and *N*-type VACCs detected single bands of ~18, ~240 and ~260 kDa, respectively, corresponding to the reported molecular weight of these proteins; (**B**,**C**) increasing amounts of protein from HP were assayed with anti-*L*-type (**B**) or anti-*N*-type (**C**) VACC antibodies. Densitometric analysis showed a linear increase in OD with respect to total protein amount; (**D**,**E**) Equal amounts of protein from HP, ONP or NB were assayed with anti-*L*-type (**D**) or anti-*N*-type (**E**) VACC antibodies. As a control of secondary antibody specificity, omission of the primary antibodies showed no ECL-signal (middle panels of (**D**,**E**)). Bovine serum albumin was used as an external load control for these experiments (lower panels of (**D**,**E**)).

Reaction of antibodies against OMP and either *L*- or *N*-type VACC was shown by immunoblotting (Figure 1). The anti-OMP antibody detected a single band of ~18 kDa. The anti-*L*-type channel antibody recognized a single band of ~240 kDa and the anti-*N*-type channel detected a main protein band of ~260 kDa (Figure 1A). Increasing amounts of total protein obtained from hippocampus and loaded in the gels showed a linear increasing in OD intensity of the bands recognized by either the *L*- or *N*-type channels (Figure 1B,C). Antibody reactivity with proteins of cloned neuronal precursors extracts was compared with immunoreactivity of proteins in rodent hippocampal tissue, and N1E-115 cell homogenates. The anti *N*-type and *L*-type calcium channel antibodies recognized a protein band of similar molecular weight in extracts obtained from different cells. However, tissue-specific differences were observed and major detection was observed in hippocampus tissue, while a slight band was seen in NIE-115 cell extracts. Specificity of secondary antibodies was verified by incubation with a solution without the primary antibodies; as shown in figure 1 there were no signal bands in this condition (Figure 1D,E).

### 2.2. Immunodetection of OSN, Precursor Cells and VACC

**Figure 2:**
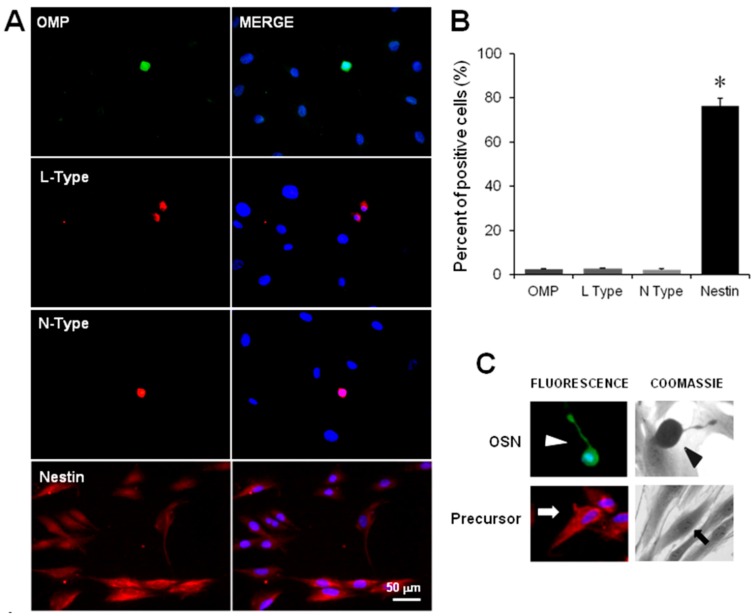
Immunodetection of olfactory sensory neurons, precursor cells and channel proteins in cultured cells obtained by the cloning assay. Cells in passage 20 were cultured during three days. OSNs were detected with an antibody against the OMP. *N*- and *L*-type channels were stained with an antibody directed against the pore-forming α-subunits of these channels. Precursor cells were detected with an anti-nestin antibody. (**A**) shows representative images of cells positively stained to OMP, *L*- and *N*-type channels and nestin. The graph in (**B**) depicts the percentage of positive cells counted in 10 randomly selected fields by triplicate: OMP(+) cells: 2.9% ± 0.5%; *L*-Type(+) cells: 1.9% ± 0.3%; *N*-type(+) cells: 3.1% ± 0.7%; Nestin(+) cells: 76.4% ± 3.5%. For negative control, the incubation of cells with each primary antibody was omitted (Supplementary Figure S1); (**C**) representative images showing an OMP(+) cell in green, and a nestin(+) cell in red detected by immunofluorescence. Nuclei were stained with DAPI and shown in blue. The arrowhead signs a cell stained with Coomassie blue that shows the typical morphology of the OSN. The arrow signs a spread flat cell. Mean and Standard error (SE) were plotted and data were compared with non-parametric ANOVA test and a Student-Newman-Keuls method. * *p* = 0.029.

The cellular stage of neurodevelopment was identified by immunofluorescence staining of specific markers, *i.e.*, the protein nestin was detected to identify precursor cells and the protein OMP to distinguish OSN. In addition, immunodetection of the α subunit of *N*- or *L*-type Ca^2+^ channels was performed. In these experiments, the proportion of OMP positive stained cells [OMP(+)] was similar to the proportion of cells labeled with the *N*- or *L*-type channel antibodies, but was significantly lower regarding the population of nestin(+) cells (Figure 2A,B) (non-parametric ANOVA, *p* = 0.029). Figure 2A (right panel) shows the nuclei counterstained with DAPI, which made possible the assessment of the proportion of each cell population.

Due to the impossibility to perform electrical recording in immunostained fixed cells, we identified OSN by their typical morphological features, characterized by a round or ellipsoidal soma from which a dendrite with a knob at its end is projected. Similarly, precursor cells showed a spread flat cytoplasm without clear projections. This characteristic morphology of OSN (OMP+) and precursor cells (nestin+) was confirmed by immunofluorescence staining (Figure 2C).

**Figure 3:**
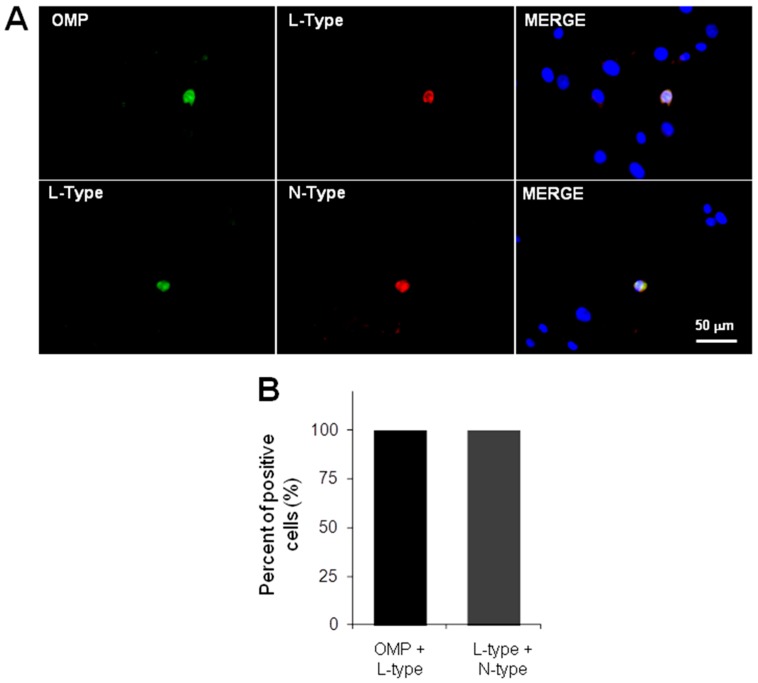
*L*- and *N*-type Ca^2+^ channels detection in olfactory sensory neurons. Cells in passage 20 were cultured over three days. (**A**) Representative images with simultaneous staining of OMP and the *L*-type channel α-subunit and of both *L*- and *N*-type channel α-subunits; (**B**) graph shows that *L*-type channels were detected only in OMP(+) cells, and in addition, both channel types were co-detected. For negative control, the incubation of cells with the primary antibodies was omitted (Supplementary Figure S1).

Thus, to explore whether these channels are detected specifically in OSN but not in precursor cells, simultaneous double-staining procedures were performed. Representative images show that the *L*-type channel α subunit was found only in OMP(+) cells (Figure 3). Additionally, images show that both *L*- and *N*-types of Ca^2+^ channels were co-detected (Figure 3).

Taken together, immunofluorescence results suggest that both of the VACC pore-forming proteins detected were specifically expressed in OSN.

### 2.3. Forskolin Induced an Increase in the Concentration of Intracellular Ca^2+^

**Figure 4:**
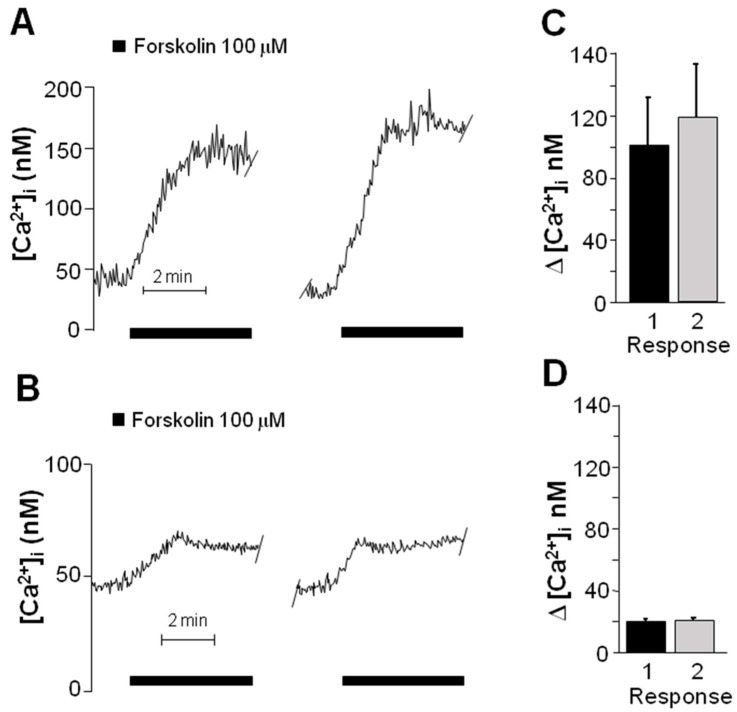
Forskolin-induced response in neuronal cells isolated from the human olfactory epithelium. Cloned cells in passage 20 were plated in round coverslips treated with rat collagen and cultured for three days. Intracellular free Ca^2+^ concentration increase was elicited with a perfusion of forskolin and measured by microfluorometry using Fura-2. (**A**) Intracellular Ca^2+^ concentration measured in OSNs; (**B**) intracellular Ca^2+^ concentration measured in Neuronal Precursors (NP). Two stimuli of forskolin were applied with an inter-stimulus interval of 15 min; (**C**) OSN; and (**D**) NP. Comparison between the amplitude of the responses obtained with both forskolin stimulations. Mean and SE were plotted and data were compared with a paired Student *t*-test; statistical differences were not detected between the responses in each group ((**C**) *p* = 0.718 and (**D**) *p* = 0.938).

Intracellular free Ca^2+^ concentration was determined by measuring the fluorescence emitted by the Fura-2/Ca^2+^ complex to study the functioning of VACC in cells previously identified, as described above, as OSN or precursors (*n* = 5). Baseline concentration of Ca^2+^ was 50 ± 13 nM and no statistical differences were detected between OSN and precursors (Student *t*-test, *p* = 0.62). Furthermore, incubation with forskolin induced an increase in intracellular Ca^2+^ concentration in both types of cells; however, the response was five-fold higher in OSN (Figure 4A) than in precursor cells (Figure 4B), and significantly different (Figure 4C,D) (Student *t*-test, *p* = 0.027). In all experiments, two stimuli of forskolin were applied with a 15-min inter-stimulus period, and no statistical differences were found in the response amplitude (Figure 4C,D) (Student *t*-test, in Figure 4C *p* = 0.718 and in Figure 4D *p* = 0.938). The forskolin-induced response was dependent on the neurodevelopmental stage of stimulated cells (undifferentiated precursors or mature neurons).

**Figure 5:**
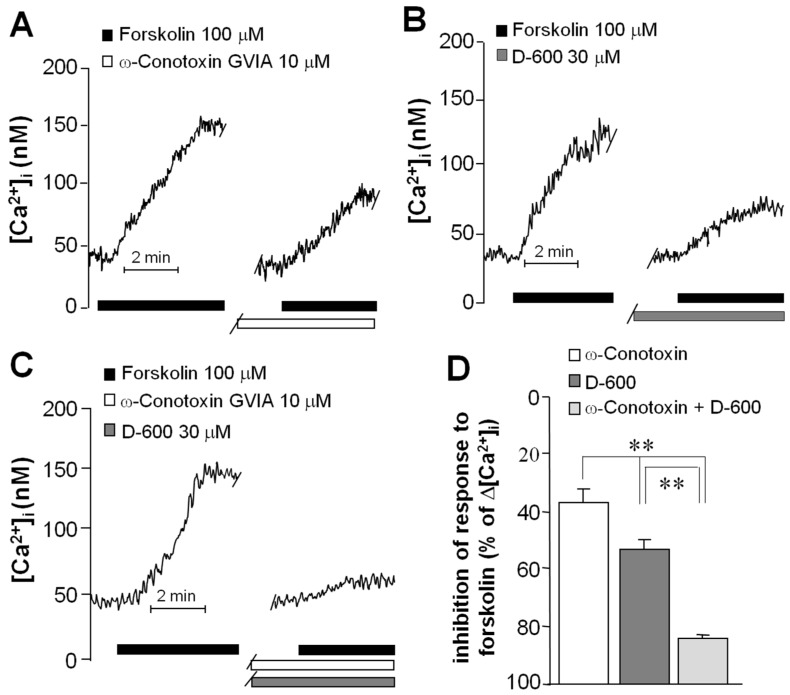
Participation of VACC in the forskolin-induced response. Microfluorometric measurements were performed in OSNs. Increase in intracellular Ca^2+^ concentration was induced with forskolin and the participation of specific types of VACC was evaluated by blocking the second response with ω-Conotoxin for *N*-type channels (**A**); D-600 for *L*-type channels (**B**); and a mix of both blockers (**C**); The contribution of each channel type was determined considering the amplitude of the first response as 100% (**D**). Mean and SE were plotted and data were compared with a one-way ANOVA test and a *post hoc* Tukey test. ** *p* < 0.001.

To determine VACC involvement in the forskolin-induced intracellular Ca^2+^ increase, OSNs were selected for recording and the specific Ca^2+^ channel blockers ω-Conotoxin (to block *N*-type channels) and D-600 (to block *L*-type channels) were used. Cells were perfused with the blockers 5 min prior to the second stimulation with forskolin, and the amplitude of this response was compared with the first to determine the contribution of each channel type to the total increment of intracellular Ca^2+^. In cells perfused with ω-Conotoxin (*n* = 5), the amplitude of the second response was reduced by 43% regarding the first (Figure 5A). Perfusion with D-600 (*n* = 5) reduced the second response by 55% (Figure 5B), and the mix of both blockers (*n* = 5) blunted the response by 89% (Figure 5C). Significant differences were obtained between the groups (ANOVA and Tukey test, *p* < 0.001) (Figure 5D). These results indicate that the forskolin-induced increase in the intracellular free Ca^2+^ concentration mainly depends on the opening of both types of VACC. Moreover, because the mix of blockers acts in an additive manner, it could be assumed that Ca^2+^ flows through both *N*- and *L*-channel types present in the same cell.

### 2.4. Electrophysiological Recording of VACC-Dependent Currents

**Figure 6:**
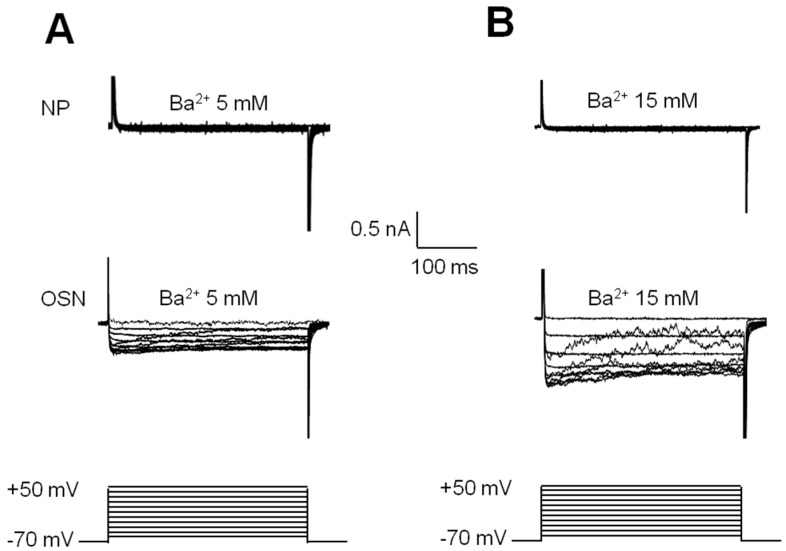
VACC-dependent currents evoked by depolarization steps in neuronal cells obtained from the human olfactory epithelium. Cells in passage 20 were cultured for three days and VACC-dependent currents measured by the whole-cell patch-clamp technique, employing Ba^2+^ to replace Ca^2+^ as the inward charge, and with a holding potential of −70 mV. (**A**) representative example of the effect of 5 mM Ba^2+^ perfusion in neuronal precursors (NPs) or in OSNs; in OSNs, sustained inward currents were evoked with depolarizing steps ranging from −60 to +50 mV; (**B**) representative recording perfusing 15 mM of Ba^2+^ in NPs or in OSNs. In NPs, no changes in current were elicited with the same protocol of steps with 5 or 15 mM of Ba^2+^.

**Figure 7:**
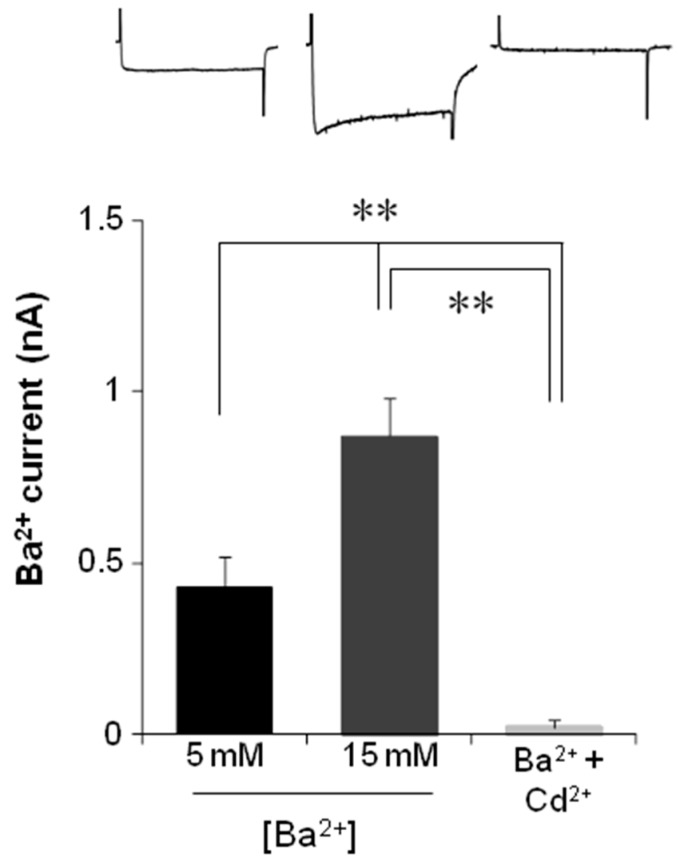
Barium current through VACC evoked by voltage steps in olfactory sensory neurons. To corroborate that currents in OSN were dependent on Ba^2+^ entry through VACC, the barium concentration ([Ba^2+^]) in the extracellular solution was increased. Additionally, Cd^2+^ was perfused to block VACC. In the upper graph, representative recordings obtained at the 0-mV step are shown. Mean and SE were plotted and data were compared with a one-way ANOVA and a Tukey test. ** *p* < 0.001.

OSNs or neuronal precursors were selected for recording by the whole-cell patch-clamp technique replacing Ca^2+^ with Ba^2+^ as the inward charge and with a holding potential of −70 mV. In the group of OSN (*n* = 15), depolarizing steps evoked sustained currents by Ba^2+^ entry (Figure 6A). In contrast, when the same protocol of steps was applied to precursor cells (*n* = 30), no Ba^2+^ currents were evoked (Figure 6B). To confirm that the evoked current was dependent on Ba^2+^ entry, OSNs or precursors were perfused with a solution containing 15 mM of Ba^2+^ instead of the 5 mM used previously. This change in Ba^2+^ concentration induced an increase in the inward current evoked by voltage steps in OSN but not in precursors, as expected (Figure 6). Additionally, perfusion of cells with a solution that contained 5 mM Ba^2+^ and 100 µM Cd^2+^ blocked the Ba^2+^ entry in OSNs (Figure 7). These experiments showed that depolarizing steps evoked a Ba^2+^ inward current through VACC in OSN. However, this response was not evoked in precursor cells.

**Figure 8:**
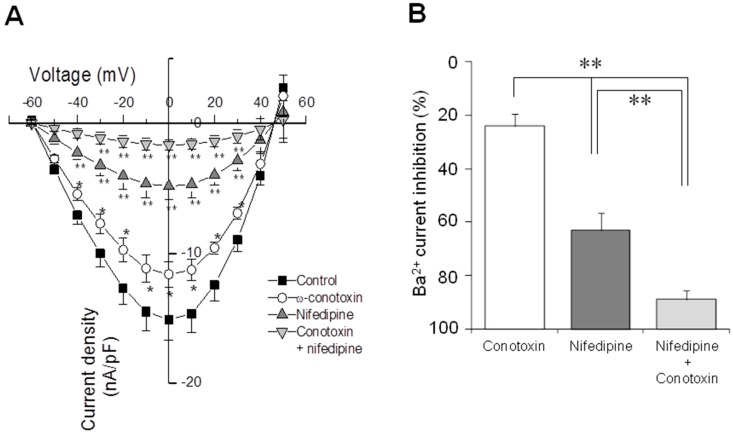
Contribution of *N*- and *L*-type channels to Ba^2+^ current evoked by depolarization steps. Electrophysiological recording by patch-clamp was performed in OSNs. Ba^2+^ current was blocked with either ω-Conotoxin to study the *N*-type current or with Nifedipine to block the *L*-type current. In addition, perfusion with the mix of both blockers was assessed. In (**A**), a current-voltage (*IV*) graph is depicted. Reversal potential and input resistance were similar in all pharmacologic conditions. Current density between groups was analyzed with a one- way ANOVA followed by Dunnett’s multiple comparison tests (* *p* < 0.05 and ** *p* < 0.01 when compared with control group); (**B**) shows a comparison of the remnant current after blocking procedure. Mean and SE were plotted and data were compared with a one-way ANOVA test and a Tukey test. ** *p* < 0.001.

The contribution of each VACC type was studied by perfusing OSN with specific blockers. With perfusion of ω-Conotoxin (*n* = 5), the amplitude of the Ba^2+^ current was reduced by 25%, and with Nifedipine (*L*-type channel blocker), a reduction of 66% was observed (*n* = 5) (Figure 8). Simultaneous perfusion of cells (*n* = 5) with both channel blockers resulted in a reduction of 88% in total Ba^2+^ current (Figure 8). These results together with those obtained by microfluorometry, suggest that both types of functional Ca^2+^ channels are present in the same OSN, and that both *N*- and *L*-type currents are predominant in total Ba^2+^ current evoked by depolarization steps. The reversal potential and the voltage at which the half of maximal current appeared were calculated by extrapolation. Comparison of the reversal potential showed no statistical differences between experimental groups (Control = 46.7 ± 1.2 mV, Conotoxin = 48.2 ± 0.9 mV, Nifedipine = 47.6 ± 1.7 mV or Conotoxin + Nifedipine = 47 ± 1.5 mV; ANOVA, *p* = 0.87). In a similar fashion, there were no differences in the potential at which the half of the maximal current was reached (Control = −38.1 ± 1.4 mV, Conotoxin = −37.4 ± 2.1 mV, Nifedipine = −36.2 ± 1.5 mV, Conotoxin + Nifedipine = −36.5 ± 1.7 mV; ANOVA, *p* = 0.8). Data suggests that channel blockers act specifically upon the *L*- and *N*-type channels and that in these conditions of recording no other cationic current was involved.

## 3. Discussion

In this work, we found that both *L*- and *N*-type VACC presence and functioning is dependent on the developmental stage of neuronal cells obtained from the human olfactory epithelium. Pore-forming α subunits from both *L*- and *N*-type channels were co-detected in OMP(+) cells (OSN) but not in precursors. Regarding VACC functioning, OSN respond to forskolin with a high entry of Ca^2+^ and depolarization steps evoked a specific Ba^2+^ inward current, whereas the forskolin-induced response observed in precursor cells was low and independent of VACC opening.

Specificity of the antibodies used in this study was corroborated by Western blot analysis of proteins separated by SDS-PAGE and molecular weight determination of the recognized bands. Our results showed a single ~18 kDa band when membranes were incubated with the anti-OMP antibody. As well, the anti-*L*-type VACC antibody recognized a single ~240 kDa band, while the anti-*N*-type VACC antibody detected a main ~260 kDa band. Evidence reported previously indicates that specific antibodies against these VACC recognized single bands of similar molecular weights [23,24,25,26,27]. In addition, when increasing amounts of total protein were loaded in the gels, the optical density of the bands showed a linear increase. Moreover, abundant expression of VACCs has been reported to occur in the rodent hippocampus, while decreased levels of these channels were described by electrophysiological recording in neuroblastoma cells [28,29]. Results found in our study concur with these findings since strong signal bands were detected in extracts of rodent hippocampus. Meanwhile, thinner bands were observed in extracts of neuroblastoma cells. Besides, specificity of the secondary antibody was determined omitting the incubation with the primary antibody. Together, data indicate that primary and secondary antibodies used in this study are specific.

Several reports have established that isolated OSN are bipolar cells that exhibit characteristic morphological features, such as a round or ellipsoidal soma from which a dendrite with a knob at its end is projected. In contrast, precursor cells are flat and without clear projections [7,30,31,32]. Moreover, protein markers have been used to distinguish these cellular stages in culture: the protein OMP to detect OSN [33,34] and the protein nestin to recognize precursors [35,36]. In this study, we immunodetected the pore-forming α subunits of *L*-type channels simultaneously with the OMP, and both types of channels (*L* and *N*) were also co-localized in OSN. In addition, we demonstrated that the culture obtained by cloning assay was enriched with precursor cells since they were labeled with the anti-nestin antibody. Moreover, in our hands, some cells spontaneously differentiated into OSN as has been observed in other reports by anti-OMP antibody staining [31,37,38]. These results indicate that expression of VACC is a mechanism that might be acquired by OSNs along their developmental process.

To study the functioning of the VACC in OSN, the influx of Ca^2+^ was analyzed by microfluorometry and electrophysiological recording. These cells were stimulated with forskolin to increase cAMP levels and to induce VACC opening. The forskolin-induced increase in intracellular Ca^2+^ concentration was five-fold higher in OSN than in precursor cells, in which a minimal change in intracellular Ca^2+^ was detected. To confirm this result, the patch-clamp technique was utilized to discard the possible involvement of VACC in the precursors’ response. No Ba^2+^ current could be recorded in precursors by depolarization steps, which strongly suggests the lack of functional VACC in these cells in accordance with the immunofluorescence results.

Previous reports have indicated that functional voltage-activated channels are not present in neuronal precursors isolated from the brain, bones or adipose tissue [39,40,41,42,43]. These channels, which are Ca^2+^- or Na^+^-permeable, allow for mature neurons to generate the neural coding [44]. The expression of voltage-activated channels in culture starts in the presence of trophic factors that induce the differentiation of precursors into specialized neurons, suggesting that voltage-activated channels and mechanisms of excitability could be acquired by neurons along their developmental process [15,16]. The results obtained in this work are in agreement with this hypothesis.

We previously described that in primary cultures obtained from the olfactory neuroepithelium of patients with schizophrenia, the *L*-type VACC-dependent current was 50% lower than in cultures derived from healthy subjects [30], suggesting functional alterations of VACC in neurons from neuropsychiatric patients. SZ has been associated to neurodevelopmental alterations, thus, as a first step to address the study of a possible association between VACC functional expression and neurodevelopment, in this study we characterized these channels in cloned cells obtained from a healthy subject. This strategy allowed us to have a genetically homogeneous population of neuronal precursors that produce more robust responses. In this regard, our culture was derived from a single, mitotically-active precursor cell obtained by a standard cloning procedure [22]. The lack of exogenous trophic-factor incubation implies that recorded OSN had spontaneously matured under our culture conditions. This is in accordance with Hahn and co-workers [45], who observed that in the culture of olfactory epithelium-derived precursors, some OMP(+) cells were co-stained with an antibody against BrdU, a DNA-replication label, indicating that proliferation was followed by spontaneous differentiation into mature OSN. Taken together, our results indicate that recorded OSN matured spontaneously under culture and additionally, that functional VACC were acquired along the specialization process that occurs under our conditions.

VACC in OSN are involved in the propagation of sensory receptor potentials in response to activation of the olfactory transduction pathway [7]. In this regard, odorants induce activation of adenylate cyclase and consequently raise the cAMP concentration. The binding of cAMP to nucleotide-operated Ca^2+^ channels [46] leads to an increase of intracellular free Ca^2+^ that, in turn, induces the opening of Ca^2+^-gated Cl^−^ channels [47]. The outward Cl^−^ current depolarizes the transmembrane potential of the OSN and this reduction in polarity leads to the opening of VACC, which amplify sensory receptor potentials to propagate across the dendrite and the soma of the cell [32,48]. In this work, OSN were perfused with forskolin, a specific adenylate cyclase stimulator. A plausible explanation of our results is that the forskolin-induced increase in intracellular free Ca^2+^ was achieved by the activation of the olfactory transduction pathway, which resulted in downstream opening of VACC, as has been observed elsewhere in OSN obtained from other mammals [49,50]. On the other hand, the subtle forskolin-induced response observed in precursor cells could be related with the opening of nucleotide-operated Ca^2+^ channels or with another transiently expressed mechanism; however, further research is required to elucidate this hypothesis. In this regard, in patients with SZ there is a decreased perception of odorants which suggests an impairment of this signaling transduction pathway [6]. Our results support the usefulness of olfactory neuroepithelial-derived clonal cultures to evaluate VACC functioning. Future studies would address the in-depth biochemical and functional analysis of these channels and other elements of the odorant signaling pathway in neuropsychiatric patients’ samples.

Likewise, data obtained in this work is in agreement with the hypothesis that acquisition of excitability during the differentiation process of the sensory neurons from the olfactory epithelium is similar to the process occurring in neurons from the CNS, in which differential expression of channels and progressive acquisition of functionality have been observed [15,16]. The embryonic origin of OSN is similar to that of CNS neurons [51]; therefore, cultures of precursor cells obtained from the human olfactory neuroepithelium could be proposed as a suitable model to study the role of VACC in the neurodevelopment process. There is evidence of VACC participation in the development of specialized neuronal functions such as synaptogenesis or neurotransmitter synthesis and release [52,53]. Actually, the culture of olfactory neuronal precursors has allowed characterizing alterations in microtubule cytoskeletal arrangement in samples from neuropsychiatric patients [54]. Thus, this culture is useful to study alterations in the differentiation process at the cellular or subcellular level in neuropsychiatric disorders.

In conclusion, our data support the hypothesis that the mechanism for generating the sensory receptor potentials by which the encoding in OSN could be achieved, is a feature acquired by newborn neuronal cells during their specialization process. Further studies could consider VACC as markers of acquired excitability, in addition to the OMP, which has been extensively accepted as a structural marker of the mature OSN stage.

## 4. Materials and Methods

### 4.1. Enriched Culture of Human Olfactory Precursor Cells

Written consent was obtained from the donor which was a female subject with no antecedents of neuropsychiatric diseases. The protocol was approved by the Institutional Ethical Committee and the study was conducted in compliance with the Declaration of Helsinki for Medical Research Involving Human Subjects. Olfactory neuroepithelial cells were obtained by exfoliation of the nasal cavity with a special brush following the protocol described in detail by Benítez-King *et al.* [8]. Briefly, cells adhered to brush bristles after exfoliation were immersed in Dulbecco’s Modified Eagle Medium/Nutrient Mixture F-12 (DMEM/F12) culture medium supplemented with 10% Fetal Bovine Serum (FBS), 2 mM of l-Glutamine, and 1% of Streptomycin-Penicillin; these were then mechanically dissociated, passing the fragments several times through pipette tips of different diameters and seeded in a multi-well chamber. Adherent cells were cultured at 37 °C with 5% CO_2_ and aliquots cryopreserved in liquid nitrogen after each passage.

Cells from a confluent culture in passage 3 were detached enzymatically with a 0.25% trypsin-EDTA solution dissolved in PBS (in mM): 137 NaCl, 2.7 KCl, 10 Na_2_HPO_4_ and 2 KH_2_PO_4_, pH 7.3, and the number of cells was determined with a hemocytometer. Cells were diluted with a supplemented medium in order to seed only one cell per well following the protocol for establishing a cloning culture of precursor cells [22].

Wells with a single attached cell were selected and the culture medium was changed every three days. After cellular density increased by proliferation of the initial cell, detachment protocol with trypsin was used to reseed and propagate the cells in culture bottles. Although the culture derived from the initial mitotically active cell was passed over 60 times, we utilized the cultures in passage 20 or 21 to conduct the experiments as described later.

### 4.2. Antibody Characterization by Western Blot

Characterization of antibodies was performed following the experimental design described by Yunker *et al.* [55]. Extracts of rat olfactory epithelium (OE), as well as homogenates of mouse hippocampus, cloned human neuronal precursors and N1E-115 cells were prepared as described [30]. Briefly, after rodent sacrifice the OE and hippocampus were quickly dissected and placed on ice-cold lysis buffer (RIPA). Cloned neuronal precursors and N1E-115 cells were cultured in DMEM/F12 supplemented medium and scrapped out in the same lysis buffer. Tissues and cells were homogenized at 4 °C for 30 s at 40 Hz with an ultrasonicator. Total protein content was determined by the Lowry´s method [56]. OE homogenates (10 μg) were loaded into 14% polyacrylamide gels and separated by electrophoresis according to Laemmli’s method [57]. Proteins were transferred to nitrocellulose membranes following the Towbin’s procedure [58]. For OMP detection, membranes were incubated overnight with 1:1000 anti-OMP antibody (Abcam ab62144, Cambridge, MA, USA) at 4 °C. Incubation with peroxidase-conjugated secondary antibody was performed and revealed by Enhanced Chemiluminiscence (ECL) [59]. Similarly, increasing quantities of total protein of hippocampus homogenates were separated in 6% polyacrylamide gels as described previously. In addition, equal amounts of protein (20 μg) from hippocampus, cloned neuronal precursors or neuroblastoma extracts were separated and transferred to nitrocellulose membranes. VACC channel proteins were identified by overnight incubation with 1:1000 anti-CACNA1C (*L*-type) or 1:1000 anti-CACNA1B (*N*-type) antibodies from Abcam (ab84814 and ab81012, respectively). Incubation with biotin-conjugated secondary antibodies and streptavidin-peroxidase was performed and revealed by ECL. Fluorogram images were acquired with a GS-800 densitometer from Bio-Rad (Hercules, CA, USA) and optical density (OD) determined with the QuantityOne^®^ software, BioRad. Bovine serum albumin (BSA) was used as external load control for the comparison of the intensity of detection of VACC proteins in the different homogenates; 3 µg of this protein was added per 10 µg of either homogenate. In the case of the gels in which the external control was loaded, proteins were stained with Coomassie brilliant blue G-250 (BioRad).

### 4.3. Detection of Cellular Stage and VACC by Immunofluorescence

Cells were fixed with 4% paraformaldehyde diluted in PBS and permeabilized with a solution of 0.1% Tween-20/PBS; non-specific sites were blocked with a solution of 3% BSA/PBS. All primary antibodies were incubated overnight at 4 °C: an anti-nestin mouse monoclonal antibody (1:200, Millipore, Temecula, CA, USA) to stain neural progenitors [35,36], an anti-OMP rabbit polyclonal antibody (1:100, Abcam) to detect OSN [32,33], and anti-α subunits of *L*-type channel (mouse monoclonal) and *N*-type channel (rabbit polyclonal) antibodies (20 µg/mL and 1:600, respectively, Abcam) were used. Fluorochrome-conjugated secondary antibodies (Fluorescein IsoThioCyanate (FITC) or TetramethylRhodamineIsoThioCyanate (TRITC)) were incubated for 2 h at room temperature. Nuclei were stained with 20 nM 4′,6′-DiAmidino-2-PhenylIndole dihydrochloride (DAPI) for 5 min. Coverslips were mounted with PVA-DABCO^®^ (SIGMA-ALDRICH, St. Louis, MO, USA) mounting media and preparations were observed with an epifluorescence Nikon Eclipse TE-2000 microscope (Tokyo, Japan); images were acquired with a Nikon DS-2MV Digital Sight camera and processed with Nikon NIS-Elements™ software. Percentage of positive stained cells was determined by counting the number of cells stained with each antibody, and the total number of cells was inferred with the DAPI-stained nuclei in 10 random-selected fields by triplicate. For negative controls, the incubation of cells with the primary antibodies was omitted.

To determine co-expression of OMP protein and *L*-type channels or *N*- and *L*-type channel proteins, the simultaneous double-staining protocol was utilized, following the previously described procedure to fix, permeabilize and block non-specific sites. Nuclei were also detected with DAPI and cells were observed with the epifluorescence microscope. Transformed results with the arcsin function were compared by a non-parametric ANOVA test followed by a Student-Newman-Keuls method.

### 4.4. Detection of Intracellular Ca^2+^ by Microfluorometry

Olfactory epithelial cells in passage 20 were plated at a density of 12,000 cells/cm^2^ in round, 12 mm-diameter coverslips previously coated with a solution containing rat collagen and were cultured during three or four days with supplemented DMEM/F12 medium. Cells were loaded with 2.5 μM Fura 2-AM in low Ca^2+^ (0.1 mM) and, after a 1 h incubation at 37 °C and 5% CO_2_, they were transferred into a heated perfusion chamber mounted on an inverted Nikon Diaphot 200 microscope (Tokyo, Japan). Cells adhered to the coverslip were continuously perfused at a rate of 2–2.5 mL/min with Krebs solution at 37 °C (in mM): 118 NaCl, 25 NaHCO_3_, 4.6 KCl, 1.2 KH_2_PO_4_, 1.2 MgSO_4_, 11 glucose, and 2 CaCl_2_, which was bubbled continuously with carbogen to maintain a pH value of 7.4. Cells loaded with Fura 2-AM were subjected to alternating pulses of 340- and 380-nm excitation light, and emitted fluorescence was detected at 510 nm using a model D-104 microphotometer from Photon Technology International (PTI, Princeton, NJ, USA). Fluorescence was measured at a rate of 0.5 s, and intracellular Ca^2+^ concentration ([Ca^2+^]i) was calculated according to the Grynkiewicz formula [60]. The *Kd* of Fura 2-AM was assumed to be 386 nM [61]. The mean 340- to 380-nm fluorescence ratios for Rmax and Rmin were obtained by exposing the cells to 10 mM Ca^2+^ in the presence of 10 µM ionomycin and in Ca^2+^-free Krebs with 10 mM EGTA, respectively. Rmax was 6.06 and Rmin 0.39. The fluorescence ratio at 380-nm light excitation in Ca^2+^-free medium and Ca^2+^-saturated cells (β) was 4.23. Recordings were stored in a microcomputer and analyzed using data acquisition and analysis software (Felix version 1.21; PTI) [62].

For Ca^2+^-response evaluation, OSN or precursor cells were perfused with 100 μM forskolin to stimulate the synthesis of cyclic Adenosine MonoPhosphate (cAMP) and the opening of VACC. To determine the involvement of VACC in the forskolin-induced response, two blockers of specific VACC were used: 10 µM ω-Conotoxin GVIA for *N*-type channels, and 30 µM methoxyverapamil (also called D-600) for *L*-type channels. In these experiments, D-600 was used instead of Nifedipine because the latter causes interference with fluorescence measurements [63]. Blockers were incubated separately or in a mix to determine both the contribution of each channel-type to total response and to determine whether both channel types (L and N) could be evoked in the same cell.

Maximal amplitude of intracellular Ca^2+^ was measured from OSN (*n* = 5) and precursor cells (*n* = 5) and compared employing a paired Student *t*-test. To determine the percentage of contribution of both channel types, the amplitude of the first response was considered as 100%. Data in percentage were transformed with the arcsin function and compared with a one-way Analysis of Variance (ANOVA) test and a *post hoc* Tukey test.

### 4.5. Electrophysiological Recording of VACC

Cells in passage 20 were plated as described previously to detect intracellular Ca^2+^ and were cultured for three or four days with supplemented DMEM/F12 medium. To perform electrophysiological recording, cells were selected under the microscope considering the same morphological features mentioned previously. VACC were recorded by the whole-cell patch-clamp technique [64]. Briefly, cells adhered to the coverslips were submerged in a perfusion chamber (1.5–2.0 mL/min) and bathed with an extracellular solution employing Ba^2+^ to replace Ca^2+^ as the inward charge; this solution contained the following (in mM): 136 NaCl; 6 CsCl; 5 BaCl_2_; 10 HEPES; 11 d-glucose, and 10.1 niflumic acid; pH was adjusted to 7.4 with CsOH. Pipette-microelectrodes (4–6 MΩ) were utilized for recording and all experiments were performed at room temperature. Voltage clamp and voltage pulse generation were controlled with an amplifier (Axopatch 200A, Axon Instruments, Sunnyvale, CA, USA), and the electrical signals were filtered at 1–5 kHz and digitized (Digidata 1200; Axon Instruments) at a frequency of 10 kHz. Currents were analyzed with pClamp™ software (version 9.0, Axon Instruments).

The microelectrodes were filled with an intracellular solution that contained the following (in mM): 130 CsCl; 5 MgCl_2_; 10 HEPES; 10 EGTA; 3 ATP-disodium salt, and 1 GTP-sodium salt; pH was adjusted to 7.3 with CsOH. Filled microelectrodes were moved by means of a micromanipulator and a change in pressure was induced by suction. To evoke VACC, a series of depolarizing steps was applied to the cells immediately after the formation of gigaseals. Steps ranged from −60 to +50 mV in 10 mV-increments from a holding potential of −70 mV, during 500 ms, at 1 Hz.

To determine the contribution of *L*- and *N*-type channels to the total current evoked, specific blockers were used as follows: 10 µM ω-Conotoxin GVIA to analyze the *N*-type current, and 10 µM Nifedipine for *L*-type current. In these experiments, the blockers were incubated separately or in a mix. Cell capacitance was measured throughout all of the experiments. Current amplitude was quantified at 400 ms when the barium current (*IBa^2+^*) reached a steady state. To compare the contribution of both types of channels, currents were measured prior and after the perfusion of blockers, and a current-voltage (*IV*) relationship was plotted. In addition, percentage of the currents was analyzed considering 100% as the current obtained at the 0-mV depolarization step prior to perfusion of the blocker or the mix. Transformed data were compared with an ANOVA test followed by a *post hoc* Tukey test.

## Supplementary Materials

Supplementary materials can be found at http://www.mdpi.com/1422-0067/17/6/941/s1.
