# Supplementary material for: Voltage-Activated Calcium Channels as Functional Markers of Mature Neurons in Human Olfactory Neuroepithelial Cells: Implications for the Study of Neurodevelopment in Neuropsychiatric Disorders
Source: Int J Mol Sci. 2016 Jun 14;17(6):941. doi: 10.3390/ijms17060941 (PMC4926474; doi:10.3390/ijms17060941)
Supplement: Supplementary file 1 [file ijms-17-00941-s001.pdf]

Supplementary Materials: Voltage-Activated Calcium Channels as Functional Markers of Mature Neurons in Human Olfactory Neuroepithelial Cells: Implications for the Study of Neurodevelopment in Neuropsychiatric Disorders

Héctor Solís-Chagoyán, Edgar Flores-Soto, Jorge Reyes-García, Marcela Valdés-Tovar, Eduardo Calixto, Luis M. Montaño and Gloria Benítez-King

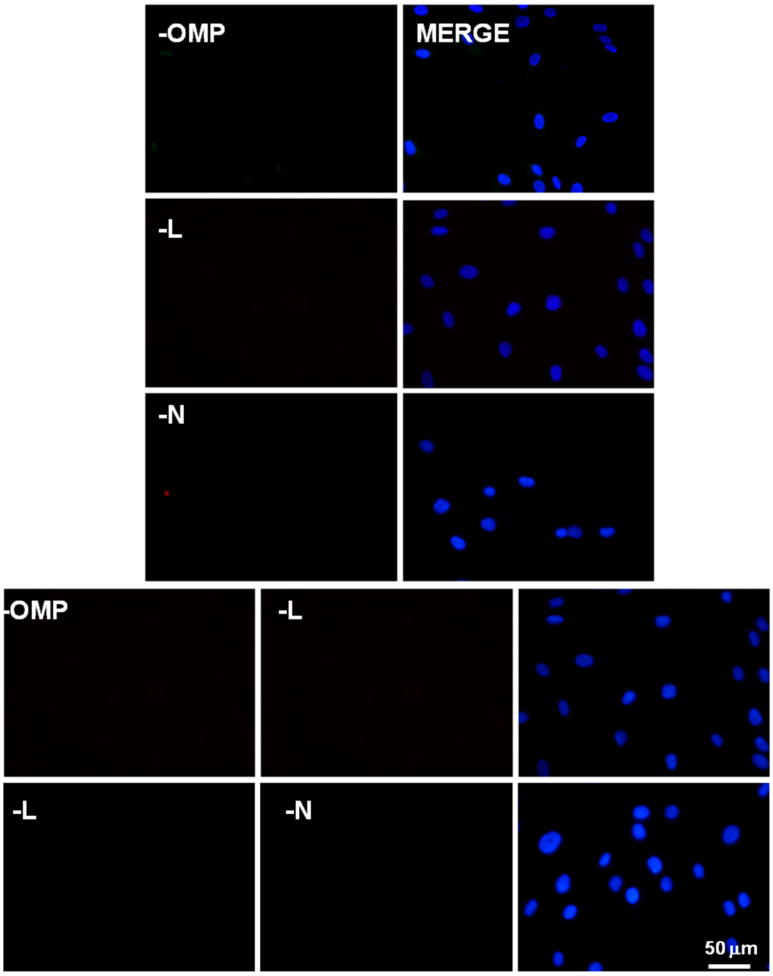

Figure S1. Negative controls for immunofluorescent detection of OMP and VACCs. In all cases, incubation of cells with the primary antibodies was omitted and nuclei were stained with DAPI. Upper panel shows representative images of preparations incubated with FITC- or TRITC-conjugated secondary antibodies corresponding to single detection of either OMP, *L*-type VACCs or *N*-type VACCs. Lower panel shows the simultaneous incubation with the secondary antibodies corresponding to a double immunostaining of OMP and *L*-type VACCs or *L*-and *N*-type VACCs.
